# The self-management of longer-term depression: learning from the patient, a qualitative study

**DOI:** 10.1186/s12888-015-0550-6

**Published:** 2015-07-24

**Authors:** Eleni Chambers, Sarah Cook, Anna Thake, Alexis Foster, Sue Shaw, Rebecca Hutten, Glenys Parry, Tom Ricketts

**Affiliations:** 1 Sheffield Hallam University, Centre for Health & Social Care Research, Collegiate Crescent Campus, Sheffield, S10 2BP UK; 2 University of Hertfordshire, Doctorate in Clinical Psychology, Health Research Building, College Lane Campus, Hatfield, AL10 9AB UK; 3 University of Sheffield, School of Health and Related Research, Regent Court, 30 Regent Street, Sheffield, S1 4DA UK; 4 Sheffield, UK; 5 Sheffield Health and Social Care NHS Foundation Trust, St George’s Community Health Centre, Winter Street, S3 7ND Sheffield, UK

**Keywords:** Depression, Patients’ perspective, Qualitative research, Recovery, Self-help, Self-management

## Abstract

**Background:**

Depression is a common mental health condition now viewed as chronic or long-term. More than 50 % of people will have at least one further episode of depression after their first, and therefore it requires long-term management. However, little is known about the effectiveness of self-management in depression, in particular from the patients’ perspective. This study aimed to understand how people with longer-term depression manage the condition, how services can best support self-management and whether the principles and concepts of the recovery approach would be advantageous.

**Methods:**

Semi-structured in depth interviews were carried out with 21 participants, recruited from a range of sources using maximum variation sampling. Interpretative Phenomenological Analysis was used by a diverse team comprised of service users, practitioners and academics.

**Results:**

Four super-ordinate themes were found: experience of depression, the self, the wider environment, self-management strategies. Within these, several prominent sub-themes emerged of importance to the participants. These included how aspects of themselves such as hope, confidence and motivation could be powerful agents; and how engaging in a wide range of chosen activities could contribute to their emotional, mental, physical, social, spiritual and creative wellbeing.

**Conclusions:**

Services in general were not perceived to be useful in specifically facilitating self-management. Increased choice and control were needed and a greater emphasis on an individualised holistic model. Improved information was needed about how to develop strategies and locate resources, especially during the first episode of depression. These concepts echoed those of the recovery approach, which could therefore be seen as valuable in aiding the self-management of depression.

**Electronic supplementary material:**

The online version of this article (doi:10.1186/s12888-015-0550-6) contains supplementary material, which is available to authorized users.

## Background

Depression is one of the commonest yet most debilitating mental health difficulties, affecting an estimated 350 million people worldwide [1]. It has traditionally been considered a time-limited condition of a single episode. However, it is now apparent that for the majority of people, incomplete recovery and relapse are more usual. More than 50 % of people, after their first episode of depression, will have at least one further episode and after the second and third episodes, the risk of relapse rises to 70 % and 90 % respectively [2]. Therefore most depression is best regarded as a chronic condition that requires long-term management [3, 4].

When considering other chronic health conditions, for example asthma, arthritis or diabetes, self-management is frequently employed and has been shown to be effective [5, 6]. However its use in depression is less widespread [7] and comparatively little is known about its effectiveness, in particular from the patients’ perspective [8, 9]. In addition, there are differing interpretations of self-management and substantial overlaps in definition with the related concepts of self-help, self-care and the recovery approach [9, 10].

Davidson defines self-management as the translation of ideas of recovery into practical tools for everyday living. In her view, this requires agency, which entails believing one can control or influence one’s environment and life [11]. Similarly, the Mental Health Foundation state:

“Self-management is about the methods, skills, and strategies we use to effectively manage our own activities towards achieving certain objectives. For those of us who live with long-term mental health conditions, this means concentrating on interventions and developing training and skills to take care of - and gain direct control over - our lives” [12].

Self-help, although well established, holds different meanings to different people. Bower et al. [13] and Gellatly et al. [14] define self-help as including therapeutic interventions administered through text, computer or other media, using individual exercises or group meetings. They state self-help encompasses activities designed to be carried out independently of contact with professionals, however, sometimes contact is involved for assessment or review. These structured activities, usually described as guided self-help, are recommended by NICE [3], and commonly delivered in the UK as a low intensity intervention in Improving Access to Psychological Therapies (IAPT) services for people with depression [15]. Alternatively other people, including those experiencing depression themselves, place more emphasis on contact with others, specifically mutual support within self-help [7]. For example, Depression UK, a national voluntary sector organisation run by people with depression, has the mission statement:

“To promote mutual support between individuals affected by or at risk from depression, with the aim of encouraging self help, recovery and personal growth” [16].

Self-care is not a term that is commonly used in mental health, however when it is there are common features with previous terms described. Gillard et al. use a broad definition of self-care that incorporates: “lifestyle strategies, social networks, and challenging exclusion, which is consistent with the recovery approach” [10]. The recovery approach involves people making sense of their experiences of mental distress in ways that allow them to preserve or reconstruct a sense of personal efficacy, agency or control, and to re-establish meaningful connections with others. However, it has to date been most closely associated with psychosis only and rarely with depression [11, 17, 18].

Although self-management and its related concepts are valued in some mental health services, many have yet to embed them in routine care and numerous barriers to implementation still exist [10, 17]. Furthermore, there has been little research to test the capacity of services to facilitate self-management as defined by those with depression or to enable such people to develop the expertise required to manage the condition.

Primarily research has focussed on approaches that involve professional input such as guided self-help [19] rather than self-management strategies that require little to no input from professionals [20]. There is greater evidence of the latter in the grey literature; however, this has rarely been used in service development, despite such strategies being available. For example, in 1999 the Mental Health Foundation recommended that:

“Mental health organisations disseminate information about the range of strategies that people find helpful, in order to assist people find and develop their own strategies and to locate alternative sources of help” [21].

Research into the self-management of depression has found that a wide range of strategies are used [8, 21–23]. Morgan and Jorm have found as many as 282 distinct self-help strategies from a literature search [24]. It has been suggested, however, that the many choices available could cause confusion:

“Pity the layperson, or for that matter the practitioner, trying to navigate the self-help morass. We are bombarded with thousands of potential resources and contradictory advice. Should we seek wisdom in a self-help book, an online site, a 12-step group, an engaging autobiography, a treatment manual, an inspiring movie or distance writing? Should we just do it, or just say no? Work toward change or accept what is? Love your inner child or grow out of your Peter Pan? I become confused and discouraged just contemplating the choices” [25].

Yet this has not been highlighted as a concern in the wider literature. Instead, the range of strategies has been shown to be of value to those with depression, in particular in the grey literature, where a holistic approach to self-management has often been described, encompassing mental, emotional, physical and spiritual factors:

“There are many different aspects of our lives that affect our mental health, and many things that can help other than the conventional medical treatments. They include: alternative and complementary therapies, hobbies and leisure, self-help activities, talking treatments and religious or spiritual beliefs” [21].

The use of a combination of strategies in this manner is known to be beneficial [26, 27]. However, there is little information available on how people go about choosing, organising and carrying out their self-management strategies. To add to the complexity, it has been found that people differ in what strategies they preferred to use, depending on their socioeconomic status, levels of support available or other personal circumstances [22, 24, 28].

This qualitative study aimed to address these knowledge gaps by exploring self-management from the perspectives of those living with depression. It aimed to understand how people with longer-term depression manage the condition, how services can best support self-management and whether the recovery approach is a useful concept. The study formed part of a programme of research based on the theme of longer-term depression within Collaboration for Leadership in Applied Health Research and Care for South Yorkshire (CLAHRC SY) and the results informed the subsequent and final stage of this wider study [29]. To allow for the variable descriptions of the concepts involved and in order to respect the participants’ own interpretations, a broad definition of self-management has been used, incorporating elements of self-help, self-care and the recovery approach.

## Methods

The study used in depth interviews and focus groups with a sample of participants who had all experienced longer-term depression. Ethical approval was obtained through the South Yorkshire Research Ethics Committee (REC 10/H1310/84) and through the Leeds East Research Ethics Committee for the additional recruitment process (REC 09/H1306/97).

### Study design

The study was grounded within a phenomenological paradigm and used Interpretative Phenomenological Analysis (IPA) because it concerned people’s perceptions of living with and managing longer-term depression [30, 31]. In health psychology the benefits of IPA have been highlighted as a method that can be used to explore the experience of individuals and to inform practice [32]. IPA uses not only the participant’s personal experiences but also the perceptions of the researcher: “the participants are trying to make sense of their world; the researcher is trying to make sense of the participants trying to make sense of their world” [33].

The study team, comprised of five people, was diverse in terms of professional and personal backgrounds. There was an emphasis on patient and public involvement throughout. Three members of the study team had experienced longer-term depression and an advisory group, made up of different stakeholders including users of services, was regularly consulted throughout the study. This is inline with recommendations and good practice guidance for user involvement in research [34]. It is believed that involvement and collaborative working can enable better recruitment, more honest data collection, minimise bias, provide a deeper level of interpretation and promote greater consideration of ethical issues [35, 36].

### Participant recruitment

The study was advertised widely to attract a diverse sample of people who had experienced longer-term depression, through local magazines and newspapers, University networks, NHS services including GPs and a mental health trust and voluntary sector agencies. The advertisement stressed that the study was interested in the different ways people had found to manage their depression, regardless of whether they had been formally diagnosed or had used NHS services. The South Yorkshire Cohort (CLAHRC SY) [37] was subsequently used to recruit older people. Recruitment was ended when a sufficiently diverse sample was achieved.

Initially participants were asked to complete a brief screening questionnaire, which provided information on demographics, participants’ experience of depression and service use. The questionnaire also asked participants whether they preferred an individual interview or focus group (see Additional file 1).

Those participants, who expressed interest in an interview and met the inclusion/exclusion criteria as far as was possible to ascertain at this stage, were invited to a brief structured diagnostic interview to verify they met these criteria. These interviews were conducted by experienced clinicians from the wider study team (Clinical Psychologists, a Mental Health Nurse Consultant and a Psychiatrist) using the MINI diagnostic framework, which was adapted to include the criteria defining longer-term depression [38].

The inclusion criteria were:

- people with dysthymia or major depressive disorder confirmed through a structured diagnostic interview (the MINI [38])
- the major depressive disorder is recurrent (three or more reported episodes) or the current or main episode is of at least two years duration.

The following exclusion criteria were applied:

- people with other mental health conditions where longer-term or recurrent depression or dysthymia is secondary to other mental health diagnoses
- people experiencing dementia
- people under the age of 18 years
- people who did not speak English
- people not living in South Yorkshire.

Purposeful maximum variation sampling was then used to obtain a diverse range of participants with characteristics relevant to the aims of the study, in order to explore both varied and shared experiences [39]. The following characteristics were used for sampling:

- age
- gender
- ethnicity
- depression (severity, length, type)
- other health conditions
- service use
- self-defined management of depression.

### Data collection

Participants were invited to attend the individual interview approximately four weeks after the MINI interview, to emphasise a clear demarcation between the two different types of interviews.

The individual interview was semi-structured and in depth. A topic guide was used, designed by the study team, which included questions on how people experienced depression and what helped them to self-manage. The guide was piloted with people with longer-term depression for evaluation and amended accordingly. It was further revised during the interview process as new themes emerged (see Additional file 2).

Three members of the study team carried out the qualitative interviews, which were audio-taped and transcribed verbatim. All participants were offered expenses and gift vouchers of £10 to thank them for their time.

During the interview process, ethical considerations were given high priority:

- after initial written informed consent, an ongoing procedure of verbal consent was employed
- the interviewers used skills to minimise any possible anxiety or distress during the interview process
- the interviews or audio-recording could be stopped at any time
- interviews were held at a place of the participants’ choosing and they could have someone with them if they wished
- positive feedback was given at the end of the interviews [40].

The majority of the interviews were conducted by two researchers with longer-term experience of depression. Although difficult to evidence, it was felt that their lived experience and associated empathy helped participants to be open and honest when recounting details of their self-management strategies, rather than feeling judged for being unusual or peculiar in any way.

### Analysis

IPA was used for the analysis [31] with each researcher analysing their own interview transcripts. These were read several times while the researcher familiarised themselves with the data. Anything of interest or significance was noted as an interpretative memo, including key words, phrases, summaries or associations. New conceptual insights were found on subsequent readings. Initial emerging themes were recorded, “to capture the essential quality of what was found in the text” [33].

**Fig. 1:**
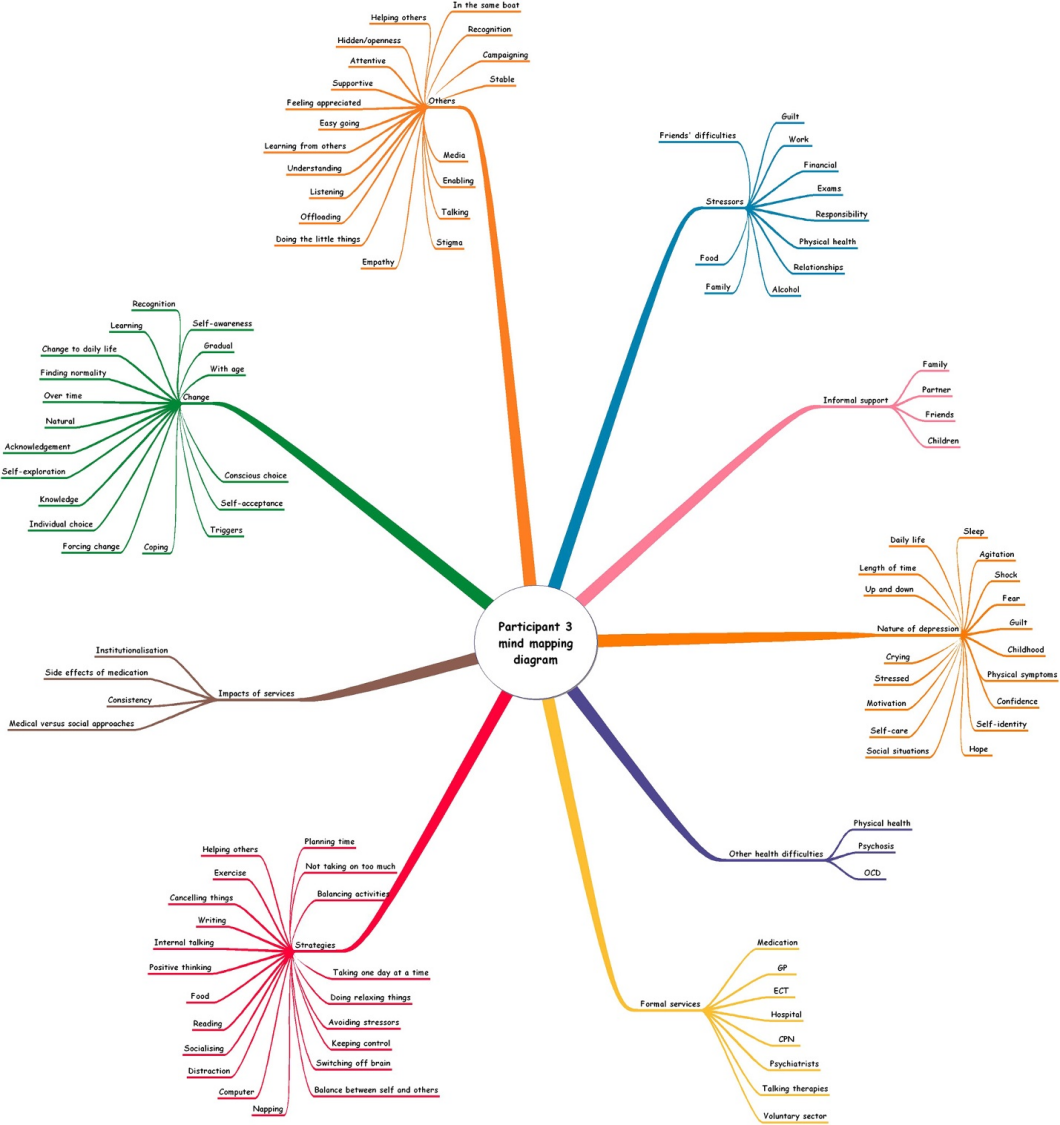
Mind-mapping diagram for participant 3. A visual representation of the super-ordinate themes and sub-themes for participant 3

A table was produced for each transcript showing evidence of the themes found. These were examined to look for connections between the sub-themes and then ordered into clusters of super-ordinate themes that related to each other. The super-ordinate themes were titled, ordered into tables (see Additional file 3) and represented visually in a mind-mapping diagram (see Fig. 1).

**Fig. 2:**
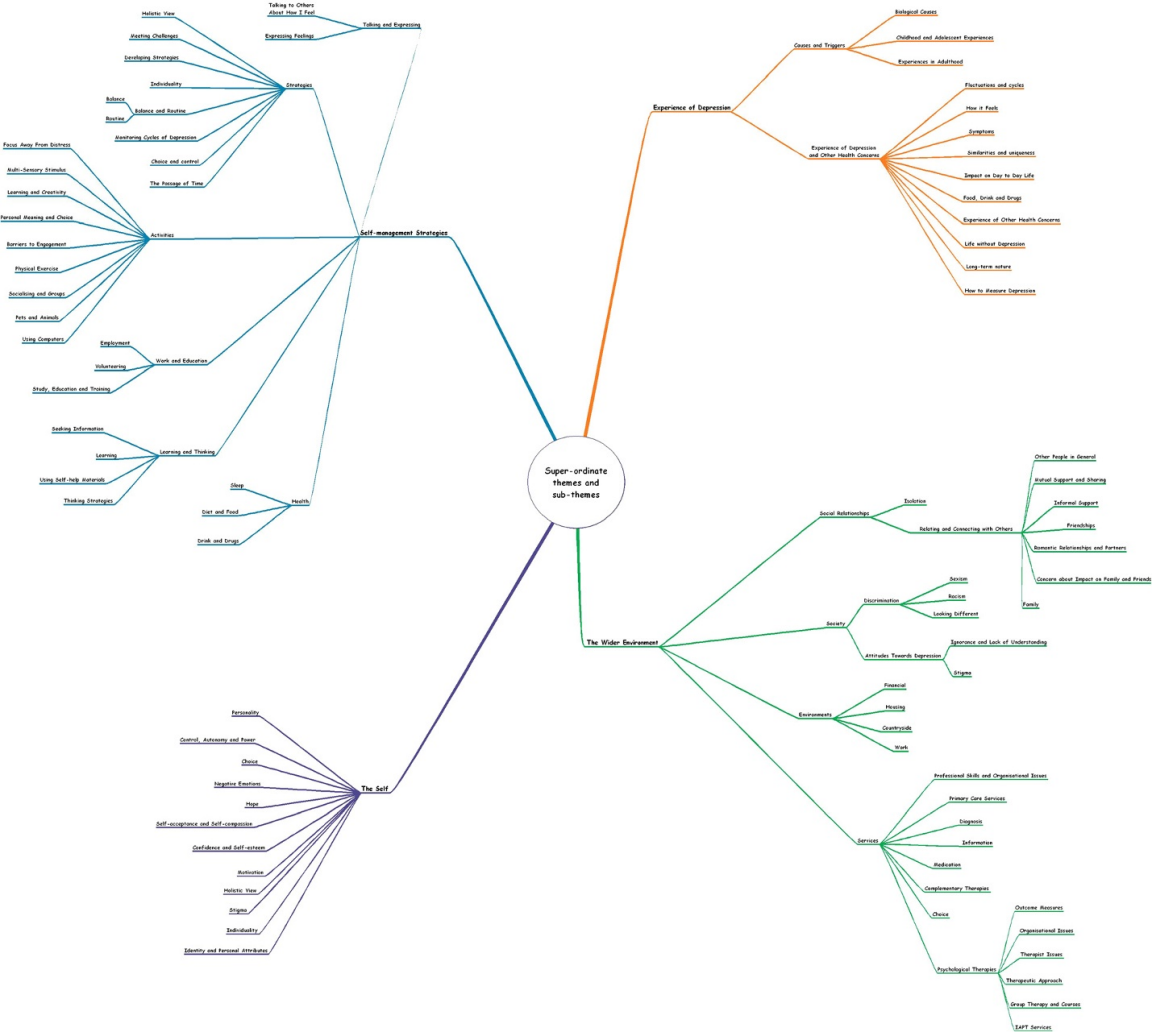
Mind-mapping diagram for all participants. A visual representation of the super-ordinate themes and sub-themes for all participants

Finally, super-ordinate themes from all the interviews were reviewed and connections between themes were considered. Patterns and cross-cutting themes were sought to suggest ideas and a structure. Also at this stage, some themes were subsumed under others and some dropped due to their lack of relevance to the research questions, resulting in one overall super-ordinate themes table and corresponding diagram being produced (see Fig. 2).

This final process of data analysis was carried out by the whole team, to ensure a variety of perspectives were used in the final stages of interpretation. The team was comprised of a lay member who had experience of longer-term depression and of using services, and academics with backgrounds in Clinical Psychology, Occupational Therapy, Public Health and Social Work, two of whom also had experience of longer-term depression. The team therefore brought to the analysis a range of views from their lived experiences in addition to their professional perspectives. Those with service user experience ensured that the prime focus was always on the participants’ unique experiences rather than what might be expected from services or from particular professionals.

Each member brought their thoughts from their reflexive diaries (see Validity and rigour) and interpretative memos to the group analysis meetings. Extensive debate and discussion ensued as members challenged each others thinking to reach a consensus and generate explanations. This lengthy and elaborate process enabled the team to use their knowledge and experiences to interpret the data at a deeper level of complexity. The team was aware that personal experience, as either a professional or service user, can bring bias as well as deep insights, therefore the raw data were continually revisited to ensure that any interpretations were grounded in the participants’ interviews rather than being personally biased embellishments.

### Focus groups

Participants for the focus groups were selected from those who had completed a screening questionnaire, had expressed an interest in attending a focus group but had not taken part in an individual interview (Additional file 1). The same characteristics were used to sample the participants as those used for the individual interviews.

A topic guide was developed that identified some of the themes emerging from the interview analyses and encouraged discussion of differing perspectives on these issues. The groups were facilitated by two non-interviewing members of the research team, one of whom was a user researcher. They were facilitated, recorded and transcribed verbatim as described by Gibbs [41]. The transcripts were analysed (one each) by the two facilitators using a standard thematic analysis [42]. This provided an opportunity for triangulation of the interview findings with an independent group of participants who had experienced longer-term depression.

### Validity and rigour

Additional strategies were employed to increase validity and rigour:

- a sub-sample of transcripts (seven, from different interviewers) were analysed independently by the two non-interviewing team members. Any arising differences were discussed and the differences incorporated into the final analyses. This triangulation process involved a range of perspectives.
- participants were offered a copy of their transcript to comment on or alter as they wished (respondent validation); one made further comments [43].
- a participatory workshop was held part-way through the study at which interim findings were presented and emergent themes discussed with a range of stakeholders, including advisory group members and other local users and carers, clinicians, service managers, service commissioners and academics. The workshop was used to both validate the interim findings and to provide an opportunity for participants to highlight other possible themes which they believed to be of importance to people in their self-management of longer-term depression.

Participants were not asked to validate the super-ordinate themes because this can be counter-productive to IPA methodology. This is particularly true in this case, where there were multiple participants, a large research team engaged in interpretation and significant time had elapsed since the interviews [44]. Therefore, other forms of validation were used by way of the focus groups and workshop.

In addition, reflexivity further enhanced the quality using a number of methods:

- reflective exercises were carried out prior to interviews to assist researchers in examining any personal beliefs connected to the research area
- reflexive research diaries were kept by each researcher, combining both a field diary and reflections [45]. These were referred to during the interview, analysis and writing stages
- all members of the research team wrote a short reflexive piece at the end of the study, highlighting those issues of particular importance to them.

## Results

### Participants

Twenty eight people returned the screening questionnaire expressing interest in an interview. At this stage, four were excluded because they did not live in South Yorkshire, one did not speak English and one had experienced depression for six months only. A further participant was excluded after the MINI interview because they had experienced only two episodes of depression.

Twenty one participants were interviewed between April and December 2011. Interviews lasted between 47 and 82 min in length and were held on University premises (16) or in participant’s own homes (5). No participants wanted anyone else to be present and all agreed to the interview being audio-taped. The characteristics of participants are presented in Table 1.

**Table 1:** Characteristics of Participants

Age	18-35	7
	36-55	8
	56-75	5
	75+	1
Gender	Female	15
	Male	6
Ethnicity	White UK	17
	White not UK	1
	BME UK	2
	BME not UK	1
Severity of depression (self-reported)	Mild	2
	Moderate	6
	Severe	7
	Moderate and Severe	1
	Mild and Moderate	1
	Ticked all 3	4
Length of depression	Average	16 years
	Range	1½-40 years
Type of depression	Dysthymia	3
	Persistent major depression	5
	Recurrent depression	13
Other health conditions	Physical health	10
	Mental health	1
	Physical and mental health	1
	None	9
Services used (some ticked more than one category)	Primary	18
	Secondary	14
	Voluntary	13
Managed depression well (self-reported)	Yes	9
	No	6
	Ticked both boxes	3
	Not responded/other	3

Two focus groups, attended by five and two participants, were held with the aim of validating the interview findings. The analyses of the groups provided data convergent with that of the individual interviews. The participatory workshop provided feedback from a range of stakeholders that also validated the interim findings and presented no new themes.

Quotes from participants have been anonymised and participants have been numbered randomly (P1, P2, etc.). Additional consent was gained from Participant 3 to allow supplementary data to be presented.

### Super-ordinate themes

**Fig. 3:**
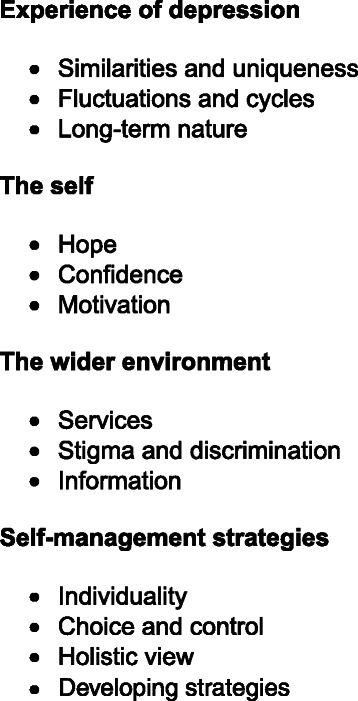
Super-ordinate themes and selection of sub-themes. The super-ordinate themes and sub-themes reported

Four super-ordinate themes were found and are illustrated with their sub-themes in Fig. 2. The detail of these findings is shown in full, with some sub-themes placed in more than one super-ordinate theme, denoting a complex inter-relationship between themes dependant on each participant’s personal experiences. Due to the richness and complexity, it is not possible to explore all the sub-themes, therefore those that were most prominent have been selected and are reported below (see Fig. 3).

### Experience of depression

This first super-ordinate theme describes how participants experienced depression and the features of most importance to them. They reported an array of symptoms: cognitive, emotional and physical. However, other aspects of depression were as important and participants relayed how depression felt to them, its impact on their lives and interaction with other health conditions. There were many similarities yet also differences, showing how each person’s experience of longer-term depression was unique.

The episodic or cyclical nature of depression was a key theme, with some participants experiencing periods in their life when they had no depressive symptoms and others continually experiencing symptoms but with the severity of the depression varying:

“Obviously within that there’s sort of fluctuations and periods where I’ve been more depressed or less depressed or probably sometimes when I’m not depressed at all.” (P16)

The fluctuations affected participants’ use of self-management strategies. Some people with cycles of depression didn’t feel the need to use self-management between episodes, others with continuous depression used strategies on an ongoing basis. Some people reported differences with each new episode of depression. The first episode in particular was cited as the worst, because at the time people did not know that the condition would improve or how to manage it.

The long-term nature of depression was often mentioned and several people felt it was important to accept that they may always experience the condition and that, rather than hoping it would go away, they needed to develop ways of managing it. Others thought it was useful to remember that their depression can become less severe:

“Well, I guess the fact that I have got better before, and that I know that people can get better and it’s not, it’s not like something that’s like incurable or whatever in that sense. I guess that is a helpful thought.” (P7)

### The self

Within this super-ordinate theme participants described how aspects of themselves were powerful agents in their management of depression. Key features were hope, confidence and motivation.

Despite many participants reporting a loss of hope and purpose in life when experiencing depression, they also relayed how both the company of other people and engaging in activities gave them hope and meaning. Examples were being with children and other family members, religious practices and being in the countryside:

“You, you’re free aren’t you? You’re like, you know, it’s just you and the elements i’n’t it…it’s more beauty and, you know, being outside and, you know and, looking at trees and birds and hills and…I think it’s that hope thing again.” (P12)

Some people had learnt to understand and challenge their negative thinking patterns that fostered feelings of hopelessness. Many participants discussed the impact of longer-term depression on their level of confidence and self-esteem. Some were able to gain confidence over time through engaging in social activities, becoming more assertive, helping other people and through finding their own successful self-management strategies. Completing tasks brought a sense of achievement:

“Things appear brighter, increases self-esteem, I really don’t ‘ave any any self-respect, you know, at the moment I don’t ‘ave a great deal of er you know, if I finish this [training] course then I’ll probably like give myself a bit of a boost.” (P19)

Finding a positive self-identity was an important aspect of coping with depression for some. This involved self-acceptance and self-compassion in order to combat the self-criticism, self-blame and negative comparisons to others that often characterised their depression.

Participants were able to motivate themselves through selecting activities that were pleasurable, however small. This helped people to keep busy, move their bodies or do something even when feeling low, lacking in energy and wanting to stay in bed:

“Look for something that I need that’ll be er useful or er read a book er, finish reading an article or you know…any number of things just kind of, you know, whatever, it gets me doing something.” (P10)

Participants explained how motivation sometimes came from rewards and a sense of achievement, such as enjoyment in seeing a finished product. This could be strengthened by being able to give to another and be appreciated, such as one person doing jobs for a relative:

“And then again it’s so nice to come back home after that, you feel you’ve done some good and achieved something.” (P8)

### The wider environment

This super-ordinate theme illustrates how aspects of the wider environment impacted on the participants’ capacities for self-management of their depression. In particular, they recounted the impacts of services, stigma and discrimination, and the availability of information.

Although services were mentioned, the majority had seemingly little direct impact on the self-management abilities of participants, with most people citing other relationships and activities as being more influential, for example:

“One of the other girls from the group who I’d kind of, sort of been friendly with while we were talking she came and found me, I actually got more out of sitting talking to her than I did out of the entire course.” (P4)

This is despite a wide range of services having been used, including primary care, secondary mental health services and different services in the voluntary and private sectors including complementary therapies. Many participants highlighted the importance of primary care services. GPs in particular were mentioned, and while some participants felt they were lacking in knowledge and experience of mental health, they were generally considered to provide an essential central role in maintaining an overview of all services and consistency over time:

“It was having someone who was having an overview because I think previously what had happened was there were lots of individual people that you got referred to that you were seeing separately. But nobody was really bringing them together so there was duplication but no real way of bringing it together and she seemed to be able to bring the different strands together.” (P11)

Participants stressed that all professionals needed skills in listening, empathy, respect and sharing power in order to engender trust and hope. Organisational issues concerned the need to be treated as an individual, the inflexibility of services and the importance of choice, in particular regarding psychological therapies:

“I think she was the CBT person I was referred to her, got on the waiting list, an appointment came through…I said ‘I’m sorry I can’t make that appointment because it’s my first week starting a new job’ and she sounded very huffy about it. And I said, but I would like to, you know, continue on the waiting list and I never heard from her again, so she’d taken me off the waiting list, just because I said, I’d rather not have an appointment (laugh) during my first week at a new job.” (P5)

Different benefits of therapies were cited by participants, including gaining a greater understanding of themselves and the nature of depression, assisting with resolving long-standing issues such as childhood abuse, and providing tools or techniques which they could use in the future. Participants valued having someone to talk to, rather than receiving an intervention through a computer:

“Just having someone to talk to was quite nice but, you know it didn’t solve my problems but it kind of, it did help a little bit I s’pose it, again it was somebody who cared, somebody who was interested.” (P4)

The importance of early recognition of depression was raised and a few participants commented how receiving a diagnosis had helped with self-management:

“I’m probably happy that it does have a name because it has helped me, erm I’ve been able to sort of change the way that I work and things that I do to try and keep me from becoming ill again and if I hadn’t had a diagnosis or a label then that’s probably not something that would have happened.” (P1)

Social stigma was experienced by many participants, who mentioned labelling, a loss of status, for example through losing employment, and other disadvantages. Some participants felt that other forms of discrimination, including racism and sexism, further compounded their depression. People felt depression was often not understood causing them to hide it, and sometimes feel shame or internalise the stigma as a result:

“Erm a lot of people think that you are weak and you are not a coper, erm and I think you actually do believe that a lot of the time yourself.” (P2)

Several participants felt that the stigma and discrimination they experienced was a barrier to them being able to access services. Some wanted to be able to self-refer to services and others explained how they didn’t want to access services at all because it would be put on their medical records:

“If I had depression erm listed on a medical record and then when if my, erm, employer found out that I have depression then…it can become one of the excuses that they use to get me, get rid of me, so… I don’t want that kind of risk, I don’t want, I know that I should have it on it because it will get me more help if and when I need it, but I don’t want it on record.” (P14)

Participants reported a lack of information or education in a number of areas, both for themselves and other people, including their family and friends, professionals and organisations, and the general public. This included information on depression, self-management and activities or groups that may assist people. Some people said they would need guidance and support to find the right information that suited them. Others suggested having lists of what other people find useful, or signposts for where to find out more information:

“Well, I think people need to be more informed and to be able to make the choices and they need and you know, if I wasn’t aware anyway, because I was saying this like you know, I was very naive.” (P15)

Many of the participants said they searched for information by reading books or newspapers, using the internet or taking part in internet chat rooms. Others researched through academic journals. There was an understanding that people differ in the type of information and guidance that would suit them. Some people found information from others with similar experiences particularly valuable:

“I have been trying to read more about depression and about other ways that people have found have worked for them.” (P11)

Sometimes information had been introduced by a therapist or mental health professional, however this could be some time after their depression began. Participants felt there was a lack of honest information about the longer-term nature of depression and that during the first episode of depression people should be given clearer information and advice to recognise and manage future episodes:

“If I could, if I’ve been, they can be told sooner then I won’t have more to deal with and it will take me much less time to actually get it, get better to then function again.” (P14)

### Self-management strategies

The final super-ordinate theme focuses on those broader aspects of self-management of depression that are of most importance to participants. They include individuality, choice and control, a holistic viewpoint and how participants engaged in developing and maintaining strategies.

**Fig. 4:**
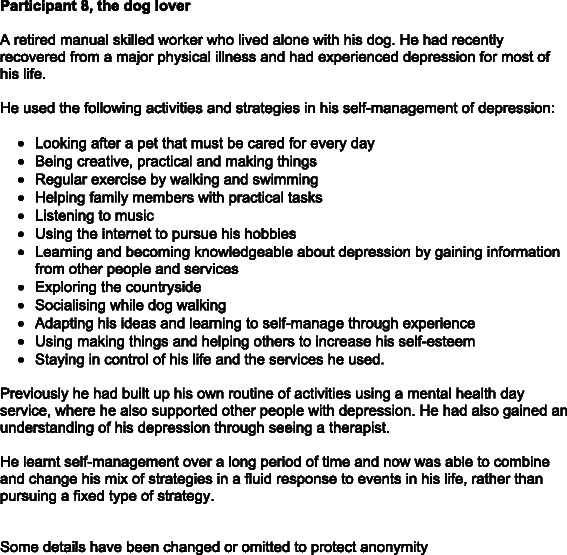
Range of self-management strategies for Participant 8. An example illustrating the diverse range of activities, self-help tools and strategies for one participant

Each participant selected a unique personalised mix of strategies, developed to suit their particular needs, interests and resources available. The mix often changed or adapted according to circumstances and over time. There was no simple recipe or package and each selection comprised a wide range of activities, self-help tools and strategies. An example of this diverse range is shown for Participant 8 in Fig. 4.

Some participants also mentioned the importance of being treated as an individual by professionals and services:

“I think you need to feel that whoever it is trying to help you is just responding to you as an individual and not through some pair of glasses that they’ve got on that isn’t about a particular way of doing things.” (P18)

Issues connected with control, autonomy and power were important to many participants. Several explained they felt less depressed when in control:

“I think it’s important to me, I think because for most of my life I didn’t feel like I was in charge really.” (P12)

Some participants explained that at times their depression could control them leaving them feeling powerless and trapped, and having to rely and trust on others to take control.

Choice was cited as being particularly important and was mentioned throughout in different contexts by many participants. This included choice about how they wanted to live their lives, what best suited their needs and having a wide selection of activities and services to choose from. Without options being available and known about, choice was not possible:

“I think we’re all different, aren’t we? And I know what I am personally and, er, I know what’s good for me, what isn’t.” (P8)

People said they chose activities that had specific positive meanings for them. For example, one participant talked about why she loved riding her own motorbike:

“It’s the speed, it’s the, it’s the unusual erm er thing about a woman on a bike.” (P15)

Several participants referred to choice with regard to services. Some felt there was a lack of choice between receiving medication and using other services and described a tendency for medication to be prescribed too readily and used as a “sticking plaster”. Others mentioned difficulties in obtaining the particular therapy they wanted:

“I mean you get referred don’t you, from your GP and it’s like pot luck really, cos you know obviously the resources aren’t limitless are they?” (P12)

Participants explained they had developed a holistic view to self-management that encompassed all aspects of their being: their minds, emotions, physicality, spirituality, sociability and creativity. This is illustrated in Figs. 1 and 4 by participants 3 and 8.

Engaging in activities was cited as an important strategy for all of the participants. The range of activities was diverse, with some holding particular value including physical exercise, green activities, socialising and being with animals. Some participants also included health services and anti-depressant medication as part of their mix of strategies. Many also had a physical health condition and a few commented on the importance of “joined-up” services for both mental and physical health.

Participants recounted how they consciously developed strategies that worked for them in their circumstances. Often they used trial and error, testing and monitoring different strategies and gradually building up expertise in what worked and how to adjust their lives to stay well. However, they reported that this was often not easy and people had to be determined and persistent:

“I s’pose it’s like trying to get a car started that’s got a flat battery, you you can’t, you’ve got to keep pushing, pushing, pushing and you’ve gotta speed it up to get it going, you know.” (P20)

Some participants felt that unresolved issues had hindered the process, others had accepted that sometimes they just needed to sit out a low period and wait until it passed or else to deal with difficulties incrementally one thing at a time.

Several participants said that addressing issues related to life events, however painful at the time, could both give a sense of achievement and enable them to develop strategies that worked for them longer-term. For example, bereavement had been a significant event for a few of the participants and this brought tremendous challenges that could be met with surprising strength despite repeated episodes of depression.

Balancing activities was found by the participants to be an effective self-management strategy. People balanced exercise and sleep; work, leisure and daily chores; stimulus and relaxation; boredom and engagement:

“I think I’ve got to that stage when I know when I’m tired that it’s time to do something else you know, get up for a walk or er ‘ave a drink summat like that”. (P20)

Participants stated that achieving a balance that suited them involved choosing between different types of activity, carefully pacing themselves, and limiting, stopping or cancelling things. Some people balanced the demands of other people and themselves with their own capacity to take on roles and responsibilities. Continual adjustment of activity was needed, depending on their mood and energy at the time:

“I watched a television programme and that was ok, but I still felt not very good at the end of it, so I had to change my behaviour and so I did some chores and still didn’t feel great so I went on the computer a bit, still didn’t feel great, so I watched another programme and then the changing of behaviour…erm, seemed to work and I got out of it…by the time I was going to bed, I felt fine.” (P5)

Many participants had found that building a routine of activities helped with self-management:

“Just gets yourself structured and you have less time to wander off and you know you’ve gotta, I don’t know, it feels good about achieving certain things each day.” (P13)

Participants had to learn to recognise their own signs of their mood becoming low and the possibility of relapsing into a period of depression. These were varied and personal to each participant:

“You’ve been in a bad mood for days, you’re crying a lot, you’re getting agitated, you’re very stressed, you’re not sleeping.” (P3)

Several participants said it was also useful to notice feeling better and capitalise on that, but warned that this can wear off and motivation can slip unnoticed. Some couldn’t recognise the signs themselves and relied on their family to alert them.

## Discussion

This study has provided an in depth exploration of the experience of living with longer-term depression and how to engage in self-management of the condition. The participants relayed a significant amount of detail about their lives and the self-management strategies used, providing rich, diverse and extensive data. The individual strategies were wide-ranging and consistent with those found in other studies of depression [8, 22]. In particular, a holistic view was reflected, that comprised the emotional, cognitive, physical and spiritual aspects of participants.

Although each participant’s experience of depression was varied the impact on participants of the longer-term nature of the condition was evident and many participants reported that the only way forward was to accept this and develop effective approaches to self-management. Four super-ordinate themes have described this process and prominent sub-themes show how each participant exercised choice and control to develop and organise their own highly individualised broad selection of self-management strategies.

This unique choice of activities and strategies was important and allowed participants to change and adapt the strategies according to their wishes and needs at any particular point in time. Although this complexity has been highlighted in the grey literature, it is still understated in academic research and healthcare guidelines, where the emphasis has traditionally been on recommending a minimal number of interventions or activities with little regard to the development or changeability of the mix of self-management strategies as attained by the participants in this study [3, 21, 22, 46].

This indicates the need for a much stronger person-centred approach to services in which the individual’s perception of their goals concerning living with depression are listened to and acted on accordingly, as has been suggested by other patient-focussed research [8, 47]. Furthermore, the importance of such an approach has been highlighted within research in primary care services, where the majority of those with depression are treated. For example, it has been shown that professionals and those experiencing mental health difficulties can hold differing definitions and values of self-help [48].

The term recovery approach also has a variety of meanings. People with a range of mental health conditions have been shown to view it differently from professionals, not as a return to pre-illness functioning, but as positive social engagement and a better quality of life, despite still possibly experiencing symptoms [49–51]. This was clearly also true for the participants in this study who experienced longer-term depression, since they relayed information about wider aspects of their lives, with an emphasis on engaging in activities and connections with other people. The development of hope, motivation, self-confidence and autonomy were found to be prominent themes, also supported by important principles in the recovery approach [52, 53]. Similarly, the participants relayed how their own personalised self-management strategies were directly linked to the way they made sense of their own experience of longer-term depression. They exercised choice and control in developing and making use of their strategies, illustrating the significance of their individuality and uniqueness [50, 52, 54].

Timeliness and transparency of information was shown to be important. During a first episode of depression, the participants wanted honest information regarding prognosis, in particular concerning the longer-term nature of depression and how self-management can be appropriately used. This is consistent with previous findings that people with depression have considerable unmet information needs regarding both understanding and managing the condition [55]. However, guidance for primary care professionals on the importance of telling people that their depression may recur and how to prevent or minimise this is minimal [3, 46]. This lack of information also applied to other people, including family and friends, and was illustrated by the stigma and discrimination experienced by participants, as has been found in other studies [9, 55].

Attitudes to the formal diagnosis of depression were mixed; some people found that receiving information on the diagnosis aided acceptance of the condition and assisted with their development of self-management. Others were uncomfortable with a diagnostic label, either fearing negative consequences or feeling stigmatised or shamed by the diagnosis. This suggests that negotiating a positive social and personal identity which incorporates depression as a constant feature was a key challenge for our participants.

Participants had used a range of formal services. GPs were highlighted as being important to provide consistency and maintain an overview of other services. Various psychological and complementary therapies were also found to be beneficial, with some providing tools or techniques that could be used on an ongoing basis. However, in general, services were not found to be useful in specifically assisting participants in the development of self-management. Furthermore, the fact that some individuals had incorporated the use of services into their repertoire of self-management strategies was not apparently due to any specific facilitation from the services.

These findings are supported by evidence from recent systematic reviews and meta-analyses of interventions for preventing the relapse or recurrence of depression, that included a variety of psychological therapies and educational interventions [56–59]. The studies showed limited evidence for the effectiveness of such interventions, however effect sizes were small or moderate, follow-up periods were limited and only a narrow range of interventions were reviewed, notably guided self-help such as computerised therapies and not the more diverse array of self-management strategies as reported in this study. Overall, this suggests that mental health services may have generally failed to develop a systematic approach to supporting and facilitating self-management and that there is greater scope for improving the ability of services to engage with this.

### Strengths and limitations of the study

The study employed a number of processes that increased validity and rigour. Triangulation was used when a service user and academic researcher from the research team carried out a secondary analysis of one third of the interviews; much agreement was found in the super-ordinate themes. Both the focus groups and workshop provided validation of the themes.

The diversity within the team provided differing interpretations during analysis. Although IPA is not usually carried out by a team of researchers, our structured reflexivity enabled us to work collaboratively to clarify and challenge our various understandings. There have been few other studies using this methodology that have taken a similar approach [35, 36]. A further strength concerns the user involvement throughout the research process, which had a positive impact in a number of ways. It influenced the design of the study, including written materials such as the topic guide (Additional file 2), analysis and writing.

Limitations of the study include the sample being restricted by the need for participants to speak English and the difficulties encountered when recruiting participants over the age of 75, despite the use of additional recruitment methods. Interviews with a selection of the health professionals and carers mentioned by participants may have provided additional data, albeit from different perspectives.

## Conclusions

This study has contributed new understanding regarding the self-management of longer-term depression from the patients’ perspective. It has highlighted themes of an individualistic holistic approach, choice and control, and the development and maintenance of self-management strategies. Although other studies have revealed a limitation in the evidence base [9, 57, 60], only a few have recently highlighted the importance of the prominent themes found here [61, 62]. Further research is needed to specifically investigate these, in particular in a primary care setting.

The study has also identified promising early findings concerning the use of the recovery approach for people experiencing longer-term depression and significant over-laps with self-management have been revealed. This is important because of the previous lack of application of the approach in those with depression. Again, additional focussed research would be beneficial.

A number of recommendations can be made for health professionals and services. Information needs to be delivered in a more transparent and timely manner. In particular, there is a need to inform people with first episode depression that the condition is often relapsing or recurrent and to support them in the early development of self-management strategies. Clarity about the diagnosis and its implications may be helpful as an aid to self-management, but there may be negative implications to this which need to be openly discussed.

Rather than offering narrowly focussed interventions such as guided self-help, it is recommended that health professionals become enablers and educators, assisting people in developing self-management strategies and sign-posting them to a broader range of approaches that reflects a more holistic view of wellbeing, based on the principles emphasised here of individuality, a person-centred approach, choice and control.

The adoption of the recovery approach would assist with providing greater choice and control for those with depression, allowing them to develop an increased sense of autonomy. Professionals need to be aware that they may have different perceptions of self-management from those experiencing depression, highlighting the importance of enabling people to develop their own individualised strategies in their own time in their own way. A partnership approach is required, that maximises the patient’s involvement and fosters an atmosphere of hope and motivation. This would include learning about the patient perspective as well as actively informing others about depression to counteract stigma and prejudice.

The need for more effective self-management practices for depression will become more urgent as demand increases and this study has shown possible benefits to both patients and services in addressing the future challenges.

## Additional files

Additional file 1: **Screening questionnaire.** Brief screening questionnaire used initially with participants.
Additional file 2: **Topic guide.** The guide used for the semi-structured interviews.
Additional file 3: **Super-ordinate themes table for Participant 3.** An example of the analysis showing the super-ordinate themes and sub-themes for participant 3.

## Abbreviations

CBT: Cognitive Behavioural Therapy
CLAHRC SY: Collaboration for Leadership in Applied Health Research and Care for South Yorkshire
GP: General Practitioner
IAPT: Improving Access to Psychological Therapies
IPA: Interpretative Phenomenological Analysis
MINI: Mini International Neuropsychiatric Interview
NHS: National Health Service
NICE: National Institute for Health and Care Excellence
NIHR: National Institute for Health Research
REC: Research Ethics Committee
UK: United Kingdom
