# Supplementary material for: The self-management of longer-term depression: learning from the patient, a qualitative study
Source: BMC Psychiatry. 2015 Jul 24;15:172. doi: 10.1186/s12888-015-0550-6 (PMC4513949; doi:10.1186/s12888-015-0550-6)
Supplement: Additional file 1: — Screening questionnaire. Brief screening questionnaire used initially with participants. [file 12888_2015_550_MOESM1_ESM.rtf]

Additional file 1. Screening questionnaire


We are carrying out a research project to find out how to improve the way the NHS helps people with longer-term depression in South Yorkshire. We want to learn about the different ways people have found to manage their depression. Self-management can mean different things to different people and can also be called self-help. If you have experience of longer-term depression, either now or in the past and whether formally diagnosed or not, we are keen to hear from you.  It doesn't matter whether you have used NHS services or not.

For this research our definition of people with longer-term depression is those who have experienced depression for 2 years or more.  We are hoping to interview as wide a range of different people as possible and completing this questionnaire will help us identify whether this study is appropriate for you. The information from this questionnaire will help us develop guidance for people who experience depression and for professionals who work with them.

Unfortunately not everyone who completes this questionnaire will be able to take part in any further stages of the study. However, it is still very useful for us to receive as many responses to this questionnaire as possible.  Please tick the appropriate boxes.

Questions about you

1)	How old are you? 

18-35		
36-55		
56-75		
76+		

2) Are you...

Female		
Male		

3) What is your ethnicity? 

White		
Mixed (white + other ethnicity)		
Asian/Asian British		
Black/Black British		
Chinese		
Other (please give details)		

Questions about your depression and services used

4) Please tick the boxes that you feel best define the severity and duration of your depression. We understand that this may be difficult to express due to the changing nature of the condition. We are interested in your personal opinion of your condition, and not how others have defined it:


Severity			Length (in years)		
Mild				
Moderate				
Severe				


5) What other health conditions do you have, if any?

	

6) Have you ever used services for your depression? 


Yes			No		

 Please tick all that you have used:

Service 		
Primary Care (e.g. GP, practice nurse, counsellor)		
Secondary Care (e.g. Community mental health team, Psychiatric hospital, Day centre) 		
Voluntary/Private Sector (e.g. Private therapist, MIND, Relate) 		

6) How well do you manage your condition?

I think I manage my depression quite well		
I don't think I manage my depression well 		

Thank you very much for filling in this questionnaire. It is much appreciated. Please complete your details below and return the questionnaire (you do not need a stamp) to:
IQuESTS, ScHARR, Freepost SF1314, Sheffield, S1 1AY

Your details:

Name:…………………………………………………………………………………………………..

Address:……………………………………………………………………………….……………….

Email address:………………………….……………………………………………………………..

Phone number:………………………………………………………………………………………..

Preferred contact time, e.g. morning or evening:…………………………………………………. 

Preferred method of contact:.………………………………………………………………….….…

WHAT ASPECTS OF THIS STUDY WOULD YOU BE INTERESTED IN TAKING PART IN?

There are two possible further stages of the study that you could be asked to participate in:

·	Being interviewed about your self-management strategies and experiences of managing depression
·	Taking part in focus groups to talk about possible self-management strategies after the interviews. 

Please indicate if you would be interested in participating in any of these: 

I am interested in being involved in interviews		
I am interested in being involved in focus groups		
I am interested in being updated about this study e.g. by receiving the newsletter		

For further information about this study please contact:
Anna Thake, Research Assistant, ScHARR, University of Sheffield, Regent Court, 30 Regent Street, Sheffield, S1 4DA; email: A.Thake@sheffield.ac.uk; telephone: 0114 222 6387.
Anna can then direct you to Eleni Chambers and Sarah Cook, Research Co-ordinators.
