# Supplementary material for: The self-management of longer-term depression: learning from the patient, a qualitative study
Source: BMC Psychiatry. 2015 Jul 24;15:172. doi: 10.1186/s12888-015-0550-6 (PMC4513949; doi:10.1186/s12888-015-0550-6)
Supplement: Additional file 2: — Topic guide. The guide used for the semi-structured interviews. [file 12888_2015_550_MOESM2_ESM.doc]

**Additional file 2. Topic guide**

**Questions, including probes**

1. Can you tell me what it’s like for you living with depression?
   - Anything connected with completed questionnaire worth asking about?
2. What helps with your depression?
   - How do these things help?
   - If people or services are involved, what could they do to help more?
3. What doesn’t help with your depression?
   - How do these things not help?
   - What could change for the better?
4. How do you know if something or someone is helping or not?
5. How could that be measured?
6. When you’re going through a particularly bad time, what makes a difference at those times?
   - What helps?
   - What doesn’t help?
   - How does your self management change at these times?
7. When things are going well, what do you do to keep that going?
8. How are things now compared to the past?
   - Are there any changes? What are they?
   - What made those changes happen?
9. Some people talk about being in charge of their life or having choices. I wonder what you feel/think about this?
   - What gives you hope in your life?
10. If you had 3 wishes to use in relation to your depression, what would you wish for?

**General prompts, can be used at any time**

- What was that like for you?
- Can you tell me anything more about that?
- And then what happened?
- Some people feel….do you feel like this?
- Is there anything else you’d like to add?
- Have you mentioned everything you’d like to?

**Specific prompts, examples of things that may help/not help**

- People, services (statutory, voluntary, private), informal support (family and friends)
- Activities, medication, diet, alcohol and drugs, pets, education, employment, complementary therapies, using a computer/the internet
- Talking, ways of thinking or believing, sense of control or choice, confidence, hope, self-esteem
