# Supplementary material for: The self-management of longer-term depression: learning from the patient, a qualitative study
Source: BMC Psychiatry. 2015 Jul 24;15:172. doi: 10.1186/s12888-015-0550-6 (PMC4513949; doi:10.1186/s12888-015-0550-6)
Supplement: Additional file 3: — Super-ordinate themes table for Participant 3. An example of the analysis showing the super-ordinate themes and sub-themes for participant 3. [file 12888_2015_550_MOESM3_ESM.doc]

**Additional file 3. Super-ordinate themes table for Participant 3**

| **Super-ordinate themes** | **Sub-themes** | **Evidence/quotes** |
| --- | --- | --- |
| Nature of Depression | up and down/fluctuating/times of day | I mean first thing in the morning when I first get up I’m not terrifically confident, I feel it's all, whoawhoawhoa and want to go back to bed (3.194)  But I do believe you can have a certain, certain control over your thoughts, um but sort of at, I don't know in the middle of the night when you’re perhaps at your, when I’m perhaps at my lowest ebb, that sort of control disappears and I get very caught up in the, in the negative stuff um. (3.248) |
|  | length of time had depression | Well I’ve been fairly well for some years, umm I still have my moments, but I've struggled all my life with depression (3.2) |
|  | affects daily life |  |
|  | poor sleep |  |
|  | agitation/bad mood/irritable |  |
|  | shock/distress |  |
|  | fear | I think one of the main things about having a breakdown is that loss of control, that the fear, terrible fear being out of control (3.204) |
|  | guilt/negative thinking | So I decided it was hopeless going through my life feeling guilty about that, you know, so, I s’pose I’ve learnt that little lesson as well, I haven't got all the answers though! (3.117)  I’m a fairly hope, well I’m, I’m fairly hopeful person but that little bit of pessimism creeps in sometimes. (3.208)  You know sometimes I say, oh but that’s going to be awful and I never wanted to do that and look at this, this has happened and my husband sort of says, oh it’ll all sort itself out, you know but I don't see it that way at the time. (3.209) |
|  | crying |  |
|  | stressed/pressured |  |
|  | motivation |  |
|  | self-care | I used to stay in bed for days on end and not, you know when I’m, when I’m in a depression phase, not washing and not looking after yourself and not keeping up with things and um, you know, like you know, like day to day life would be almost impossible, (3.174) |
|  | childhood | There were lots of problems in the family and I was in the middle of all that (3.73)  I went to a very posh girls’ grammar, I felt quite out of place because I came from a very working class background and um, that didn’t help me really over the years um and my sister was head girl, I was very jealous of her um because I was always thought of as ***’s sister, not as ***. And so there were sort of issues there. (3.216) |
|  | physical symptoms |  |
|  | confidence | When I had my first breakdown when I was 20, you emerge with not very much confidence, ‘specially if you’ve been in hospital for a while and had ECT and all this stuff, um, and you have to sort of build up your self confidence again and get back into moving, mixing with society, (3.27)  But I think through having several breakdowns and I built up a little bit more confidence after each one, I’ve ended up more confident than I was in the beginning. Which is not a way you’d recommend to anybody to build your confidence, (3.185) |
|  | self-identity | I felt as though I was completely stripped of um, um what to say, not exactly emotions but I um, confidence, no confidence, no sort of mmmm, sort of self identity really, no confidence to go out into the social situation, um and you had to build up your confidence again. (3.184) |
|  | hope | I think hope is very important and I think when you get depressed that’s what you lose sight of really, you can’t see how the future’s going to pan out or, or the future that seems to be coming together is not what you want, is, you know, is, is bad things and, and turning a corner you don't want to turn (3.207) |
|  | difficulties with social situations/social etiquette | And just walking into a room of people was the most difficult thing I’d ever done. (3.188) |
| Other health difficulties | psychosis/schizophrenia |  |
|  | OCD |  |
|  | physical health |  |
| Strategies |  | You know I learnt to sort of work round it really using various strategies. (3.5) |
|  | helping others | I, yeah I think it's with one of these, I like to be needed things again, (3.290) |
|  | planning time |  |
|  | not taking on too much | I've got friends that go out every night of the week to different classes and different things and the pictures and so on, um I, if I go out one night a week that's enough for me. (3.59) |
|  | balancing activities | You feel quite depressed because you know, it’s not um, not a good prognosis in her case and you think, you know, oh this is awful um but then I try and, I try and counteract that by thinking, right, I’ll go and tomorrow I’m gonna go swimming and I’m gonna have a peaceful day just all, you know just doing things that I like, you know. (3.169) |
|  | exercise |  |
|  | cancelling things/stopping things/slowing pace down | it seemed to sort of trigger an acknowledgement in me that I must stop, stop everything and take strategies to sort of calm down a lot (3.37)  I try and slow down the pace and talk to people, you know all the things I've mentioned really, and by doing those things I mentioned, hopefully I begin to feel better. (3.52) |
|  | doing relaxing things/finding peace | And then I go and have a coffee and read the paper and I have a great feeling of wellbeing um because of the physical exercise and it's lovely and warm in the water and you see people you know and um it just makes you feel much better. (3.157)  We do a bit of relaxation at the end and I feel, come away feeling good because I’ve sort of finally managed to relax a bit, ‘cos I'm a bit, you know, hyper all the time, intense. (3.158)  It's very sort of slow and soothing and quiet and it’s sort of non competitive, so it doesn't matter if you can’t do it, (3.229)  It’s a very peaceful environment. Very relaxing environment, you know there's no, um we live in a very noisy society, you know you go out of here and there’s sirens and people talking, mobile phones and traffic and so on and just for that hour or hour and a half it’s like a, a little oasis of calm, I read that once, that’s what they call Yoga, an oasis of calm. (3.229/230) |
|  | writing | Also I’d forgotten that writing helps me, I forgotten that’s another strategy, I wrote all about it in my first book and that was very cathartic and helped me a lot and I hope it's helped other people, um. (3.32)  Yes I think it helped me a lot writing all that stuff, that one’s about my childhood and er, family and so on (3.270) |
|  | internal talking |  |
|  | positive thinking | That’s another thing to feel guilty about, you see, if I was a mess is that why he’s like he is, that one pops up quite often, so I push it away, um but um. (3.173)  I s’pose that's one of me strategies really, I've worked out how to sort of say to myself, now look, it's no good thinking about that, or thinking in that direction, you know, (3.246) |
|  | taking one day at a time |  |
|  | switching off brain |  |
|  | reading | I particularly like contemporary novels, and if there’s an aspect of somebody suffering depression or whatever, breakdowns, whatever, so much the better, you know, I’ve read Alistair Campbell’s because he had a couple of breakdowns and I've read a couple of his novels which have been quite interesting. (3.303) |
|  | balance between self and others | My husband sometimes says, you know, you do too much for other people, you should be thinking about yourself because you’re getting stressed yourself, and he’s quite right really. You’ve got to think of number one, really, now and again, (3.142) |
|  | socialising/keeping in touch with others | The first thing I do in the morning after I’ve got dressed is turn on the computer and see if I've got any emails and my family email where sometimes they, they never used to write or phone but they email. (3.234)  I do actually write personal letters quite a lot, little, well notelets, sort of little notes if I hear that somebody’s, I don't know, somebody’s dog has died or you know, somebody’s having a hard time, I’ll send them a little hand written note and I quite, I quite like receiving those but not many people does those anymore. (3.237) |
|  | avoiding stressors | I s’pose I try and avoid stressful situations, um this isn't a stressful situation because I like talking about myself. (3.197) |
|  | keeping control/orderliness | Because everything has to be tidy, everything has to be in its place, letters that arrive have to be answered straight away. (3.201) |
|  | food | But I try and force myself to eat healthily and I think it does help your mind a lot. (3.295)  I remember the first, the second breakdown I had when I was at university living on my own in a bedsit and I was eating total junk, and I cracked up and think, looking back later and reading about how you know, your, the state of your mind and so on, depression can be affected a lot by what you eat, um I was, you know, I could look back and say well I was eating a load of junk, you know I wasn't eating healthily, but I’m very lucky to have a husband who cooks, and he cooks my tea every day and it’s always something healthy. (3.296) |
|  | computer | So I’d be lost without my computer, when it goes wrong I’m most upset (3.236) |
|  | distraction | Think about something else or read a book about something else or go for a walk or have a bath or whatever. (3.246) |
|  | napping | In the day time if I’m feeling a bit negative I sometimes go and have a sleep, I can sleep for an hour and then feel that things don't seem so bad then, (3.249) |
| Stressors | work |  |
|  | financial |  |
|  | physical health |  |
|  | responsibility |  |
|  | guilt |  |
|  | exams |  |
|  | friends’ difficulties |  |
|  | relationships |  |
|  | alcohol | And I thought it had a bad effect on me, it used to make me quite depressed, I’d be very hyper and then I’d be very depressed and I thought to myself, well my dad, who was an alcoholic used to become very morose after drinking and violent and um, after experiencing a few things myself, I decided I wasn't going to drink anymore (3.307) |
|  | food |  |
|  | family |  |
| Change | change to daily life |  |
|  | finding “normality” | I mean I would say I live a pretty normal life, whatever normal is, really what's normal, um but I didn't used to. (3.173) |
|  | recognition of depression | Um, well um, I think, mmm hard to say really, just gradually over time, something inside me would sort of click and I’d think, right, you've been in a bad mood for days, you’re crying a lot, you’re getting agitated, you’re very stressed, you’re not sleeping, I sort of suffer a lot from insomnia. (3.36) |
|  | self-awareness/self-realisation | I think um I'm very aware that when I'm starting to get ill really, and so all those different ways help me. (3.19)  I don’t know why I'm more aware, I s’pose it's just over the years sort of learn, getting to know yourself better, perhaps getting to know yourself better and what you can cope with and what you can't cope with. (3.58) |
|  | learning | After my first breakdown I thought this will never happen to me again because I’ll know it's going to happen but I didn't see it the second time, I didn't seem to be so aware (3.29)  I think I've got to know myself pretty well and I think I've learnt a lot over the years that I’m not quite a mess as I used to be. *(laughing)* (3.171) |
|  | gradual | After my breakdown at 20, as I said, I thought oh I’ll recognise, I’ll recognise it next time I'll be able to stop it happening. Now the second time I didn't really recognise it and I think the third time I recognised it but I couldn't stop it happening, so there's all those issues, really. (3.30) |
|  | over time | So, um I think I've learnt from it over the years and become more, um confident about what to do when I get like that, I think that's why I’ve been well for quite a long time (3.38) |
|  | with age | Um I think perhaps it was natural progression, so maybe as you get older you change a bit, I don’t know. (3.73) |
|  | natural | Interviewer: So, when you were younger before you’d learnt some of the strategies, is there anything that would have helped you to learn them, do you think? Could it have been done more quickly?  Um in a way no, because it's more coming to terms with the person I am and the problems I have inside my head um and only I could have adjusted to those really, and known what was going on (3.69/3.70) |
|  | triggers |  |
|  | acknowledgement |  |
|  | coping | For example when my son had a really bad accident, um I didn't actually crack up but I felt pretty awful, um and it was a big ordeal but, um I managed to get through that. (3.37) |
|  | knowledge/information | I mean when I had, I had postnatal depression in 1980 and nobody said to me, when I was pregnant, knowing I had the history I had and I had two breakdowns, nobody said to me this could happen to you, you could get depressed after the baby and you know, we’ll look out for that and you know. (3.106) |
|  | self-exploration |  |
|  | conscious choice to change |  |
|  | self-acceptance |  |
|  | individual/personal approach |  |
|  | forcing change | Baby just going to a mother and baby group or mother and toddler group was so hard that you just had to grit your teeth and do it (3.189)  You have to sort of force yourself to get up and get on with it, um I do quite a bit of forcing myself. *(laughing)* (3.194) |
| Others | keeping depression hidden/openness | I think it's best to have it out in the open, really, to help other people. (3.6)  Because like my eldest brother feels it should be in a box with a lid on, nothing should be talked about at all, well I don't think that's a very helpful attitude. (3.82)  I think the best way is to have it out in the open and talk about it as much as possible (3.84) |
|  | stigma | and I sometimes, sometimes sitting on a train and get talking to somebody next to me and I say, oh I've had 4 nervous breakdowns and they start to sort of move away from me and they’ll say it doesn't bother me, you know it doesn't bother me, it's just how I am, it's just how some people are. (3.84) |
|  | helping others/sharing stories/mutual support | But I like helping people and I like um you know, helping people with their problems, you know in a minor way with friends, I couldn't do it professionally I don't think. So. (3.143) |
|  | supportive |  |
|  | learning from others |  |
|  | attentive |  |
|  | stable |  |
|  | easy-going |  |
|  | understanding | She was marvellous and she really seemed to understand me (3.177) |
|  | doing the little things |  |
|  | off-loading |  |
|  | talking |  |
|  | listening |  |
|  | empathy |  |
|  | recognition of depression by others | I still sometimes think that my friends know me better than they do (3.150) |
|  | in the same boat/others’ stories | People can get their stories across and if that helps other people who are suffering and they read it and they say, what!, that’s just like I was, you know, it, it, it's so comforting because one of the things when I was first ill I thought I was the only person in the world who was going through that, but you know years later you realise there’s thousands, thousands more people, um who are going through similar experiences (3.90) |
|  | campaigning |  |
|  | media portrayal |  |
|  | enabling | But you know, instead of sort of right, you should do this, and you should do this, they listen and say well, do you think if you did so and so and you think, yes, you know, um but you sort of work towards it yourself really or they make it that you feel as though you’ve worked towards it yourself. (3.133) |
|  | feeling appreciated | And I have the sort of friends that’ll like send you a little note and say, you know thank you for listening to me the other day, you know. (3.147) |
| Informal support | family |  |
|  | partner |  |
|  | children |  |
|  | friends | I sometimes think I've got too many friends, trying to keep up with them all but on the other hand it's nice to be the centre of um, well like they say, friends are the family you choose for yourselves, they say don’t they? (3.149) |
| Formal services | medication | Much better, probably because of medication, I think medication that I’m on at the moment saved my life, really! 3.175) |
|  | GP |  |
|  | ECT |  |
|  | hospital |  |
|  | CPN |  |
|  | Psychiatrist |  |
|  | talking therapies |  |
|  | Voluntary sector |  |
| Impacts of services | institutionalisation | You get a bit institutionalised in hospital even if you've only been there a couple of months (3.27) |
|  | side-effects of medication |  |
|  | medical v social approaches |  |
|  | consistency |  |
